# Potential value of PTEN in predicting cetuximab response in colorectal cancer: An exploratory study

**DOI:** 10.1186/1471-2407-8-234

**Published:** 2008-08-13

**Authors:** Evangelia Razis, Evangelos Briasoulis, Eleni Vrettou, Dimosthenis V Skarlos, Dimitrios Papamichael, Ioannis Kostopoulos, Epaminontas Samantas, Ioannis Xanthakis, Mattheos Bobos, Eleni Galanidi, Maria Bai, Ioanna Gikonti, Alona Koukouma, Georgia Kafiri, Pavlos Papakostas, Konstantine T Kalogeras, Paris Kosmidis, George Fountzilas

**Affiliations:** 1 1st Department of Medical Oncology, Hygeia Hospital, Athens, Greece; 2 Department of Medical Oncology, Ioannina University Hospital, Ioannina, Greece; 3 Department of Pathology, Aristotle University of Thessaloniki School of Medicine, Thessaloniki, Greece; 4 2nd Department of Medical Oncology, Henry Dunant Hospital, Athens, Greece; 5 Department of Medical Oncology, Bank of Cyprus Oncology Centre, Nicosia, Cyprus; 6 3rd Department of Medical Oncology, Agii Anargiri Cancer Hospital, Athens, Greece; 7 Department of Medical Oncology, Papageorgiou Hospital, Aristotle University of Thessaloniki School of Medicine, Thessaloniki, Greece; 8 Department of Pathology, Ioannina University Hospital, Ioannina, Greece; 9 Department of Pathology, Agia Olga Hospital, Athens, Greece; 10 Department of Pathology, Hippokratio Hospital, Athens, Greece; 11 Department of Medical Oncology, Hippokratio Hospital, Athens, Greece; 12 Data Office, Hellenic Cooperative Oncology Group, Athens, Greece; 13 2nd Department of Medical Oncology, Hygeia Hospital, Athens, Greece

## Abstract

**Background:**

The epidermal growth factor receptor (EGFR) is over-expressed in 70–75% of colorectal adenocarcinomas (CRC). The anti-EGFR monoclonal antibody cetuximab has been approved for the treatment of metastatic CRC, however tumor response to cetuximab has not been found to be associated with EGFR over-expression by immunohistochemistry (IHC). The aim of this study was to explore EGFR and the downstream effector phosphatase and tensin homologue deleted on chromosome 10 (PTEN) as potential predictors of response to cetuximab.

**Methods:**

CRC patients treated with cetuximab by the Hellenic Cooperative Oncology group, whose formalin-fixed paraffin-embedded tumor tissue was available, were included. Tissue was tested for EGFR and PTEN by IHC and fluorescence in situ hybridization (FISH).

**Results:**

Eighty-eight patients were identified and 72 were included based on the availability of tissue blocks with adequate material for analysis on them. All patients, except one, received cetuximab in combination with chemotherapy. Median follow-up was 53 months from diagnosis and 17 months from cetuximab initiation. At the time of the analysis 53% of the patients had died. Best response was complete response in one and partial response in 23 patients. In 16 patients disease stabilized. Lack of PTEN gene amplification was associated with more responses to cetuximab and longer time to progression (p = 0.042).

**Conclusion:**

PTEN could be one of the molecular determinants of cetuximab response. Due to the heterogeneity of the population and the retrospective nature of the study, our results are hypothesis generating and should be approached with caution. Further prospective studies are needed to validate this finding.

## Background

Activation of the epidermal growth factor receptor (EGFR) is related to cell proliferation, metastasis and angiogenesis in many tumors [1]. In colorectal carcinoma (CRC) patients, EGFR is over-expressed in 75% of the tumors and its over-expression is associated with worse outcome [2]. EGFR was therefore an obvious candidate for targeted therapy in this malignancy. Cetuximab is an IgG1 anti-EGFR monoclonal antibody (moAb) that binds to the ligand-binding domain of the EGFR, leading to inhibition of its proliferative activity. Its use in the clinic has resulted in response rates (RR) of 23–25% in combination with chemotherapy and 10% as a single agent [3]. The most common side effect of cetuximab is an acneiform rash, which, however, seems to be positively associated with response and overall survival [4].

In order to avoid the unnecessary use of a costly treatment, investigators have been seeking molecular determinants of response to cetuximab, so that it could be used only on those patients that are most likely to derive a benefit. Several studies have evaluated the significance of EGFR over-expression as a surrogate marker for cetuximab response, but no such correlation has been found [5,6]. EGFR gene amplification has also been evaluated by fluorescence in situ hybridization (FISH) [7-9]. While EGFR gene amplification by FISH seemed to be associated with cetuximab benefit in one small retrospective study [9], in prospective studies and when EGFR was measured by quantitative polymerase chain reaction (PCR), its RNA levels were not associated with clinical benefit [3]. A small, recently published study confirmed the correlation of EGFR gene amplification with response to cetuximab [10].

A plausible explanation for the lack of an apparent correlation of EGFR over-expression with benefit from cetuximab is that the activation or inactivation of downstream effectors may also be necessary. One study evaluated the role of intratumoral mRNA levels of EGFR effectors, such as vascular endothelial growth factor (VEGF), interleukin 8 (IL-8), cyclin D1, and cyclooxygenase-2 (Cox-2) in predicting response to cetuximab [11]. In that study, gene expression levels of Cox-2, EGFR and IL-8 were associated with high overall survival on cetuximab, while low VEGF levels correlated with response to cetuximab.

The most compelling data so far are those that correlate the presence of K-RAS mutations with resistance to cetuximab [12]. In fact this is also the case for non-small-cell lung cancer resistance to the anti-EGFR TKIs [13]. Interesting information is also emerging in regards to the significance of VEGF and hypoxia inducible factor 1 alpha (HIFa) in the mechanism of action of cetuximab [14].

Phosphatase and tensin homologue deleted on chromosome 10 (PTEN) loss has been shown to be associated with resistance to trastuzumab [15], which is a monoclonal antibody against another EGF family receptor, Her2Neu. As Her2Neu and EGFR form heterodimers and use the same downstream signaling pathways, it was reasonable to test PTEN as a potential determinant of cetuximab resistance. This has been explored already by one group. In this recently published report immunohistochemistry evaluated loss of expressing of PTEN was associated with lack of response to cetuximab [10].

In the present **retrospective **study we collected data on all patients treated with cetuximab by the Hellenic Cooperative Oncology Group. Of note this study was performed right after the approval of cetuximab. At the time the investigators were using cetuximab off protocol in fairly advanced patients and in various lines and combinations. This, therefore, is a heterogeneous population which, however, is quite representative of common practice in Greece.

We then investigated in formalin-fixed paraffin-embedded (FFPE) tissue the protein expression and gene status of EGFR and PTEN. Association of these parameters with treatment response, progression free and overall survival from diagnosis and cetuximab initiation was explored.

## Methods

All patients with histologically confirmed, locally advanced or metastatic colorectal cancer, who had been treated with cetuximab off protocol, alone or in combination with chemotherapy, by the Hellenic Cooperative Oncology Group (HeCOG) between January 2004 and September 2005 were identified. The investigators were asked to obtain FFPE tissue from each patient for molecular analysis. Patients whose tissue was available were asked to consent for this analysis and then the tissue block was obtained and clinical data were collected.

Eighty-eight patients were identified and archival FFPE tumor samples were available for 75 patients. The material consisted of invasive or metastatic colorectal adenocarcinoma tissue, obtained from surgical resection specimens. All tissue blocks were re-cut and reviewed by the pathology team for confirmation of the diagnosis and adequacy of the material. After the evaluation 72 cases were selected for the study.

This research was carried out in compliance with the Helsinki Declaration and Ethics Committee approvals were obtained where appropriate.

### IHC

EGFR immunoreactivity was investigated using the EGFR (31G7, Zymed) mouse moAb, as previously described [16]. For EGFR staining interpretation, the proposed criteria by DakoCytomation EGFR pharmDx kit were used in correlation with the definitions by Italiano et al [17]. Sections were considered positive when ≥ 1% of the tumor cells had membranous staining above the background level and the intensity of EGFR reactivity was scored as +1, +2 and +3. Tumors with moderate (+2) and strong (+3) expression were considered as having EGFR protein over-expression [18]. Any cytoplasmic staining was considered non-specific and theses cases were evaluated as negative. Furthermore we performed IHC assays for the detection of PTEN (28H6, Novocastra, U.K.). Nuclear PTEN protein expression was evaluated according to a previously established rank scale of 0 to 2 [19]. Inflammatory and normal stromal cells were used as control markers of staining intensity. PTEN staining in tumor cells was graded as: 2, if the staining intensity was equal to or higher than that of control cells; 1, if their staining intensity was lower than that of control cells; and 0, if no staining was found in the tumor cells. Tumors with PTEN scores of 0 or 1 were considered to have PTEN loss. Cases with staining of 2 in more than 10% of the cells were considered positive for PTEN expression by IHC.

The evaluation of all IHC sections was done simultaneously by three pathologists, blinded as to the patients' clinical characteristics and survival data. All the stained slides were compared to appropriate positive and negative control sections.

### Fluorescence in situ hybridization (FISH)

Whole tissue sections (4 μm thick) were used for FISH analysis. The commercially available probes for the EGFR gene (LSI EGFR/CEP 7 Dual Color Probe, Vysis, U.S.A.) and PTEN gene (LSI^® ^PTEN/CEP 10 Dual Color Probe, Vysis) were used. The procedures were performed according to the manufacturer's instructions with slight modifications. Hybridization signals were enumerated using a Zeiss fluorescence microscope (Axioskop 2 plus HBO 100) equipped with an oil immersion ×100 objective, an appropriate filter set (DAPI, FITC/spectrum green, rhodamine/spectrum orange) and a computerized imaging system (FISH Imager™ METASYSTEMS).

### FISH analysis

The evaluation of the FISH sections was done simultaneously by two observers. The cut-off values for each probe were determined using the mean percentage of probe signals obtained from scoring 100 cells per case, from 8 normal colon tissue samples and was set at the mean plus or minus two standard deviations (SD). For the evaluation of the EGFR and PTEN gene status, 100 non-overlapping nuclei of tumor cells were also randomly selected and scored from each tumor section. Images were captured with a computer-controlled digital camera and processed with a software system (FISH Imager). The following criteria were used for the evaluation of FISH: For each FISH probe tested, the status of the chromosome (defined by the presence of centromeric probe-CEP signals) was used as control. The status of the respective gene and the ratio gene probe/centromeric probe was calculated. FISH patterns were considered normal when the ratio of the gene copies/chromosome number in each case was from 0.9–1.2 for EGFR [20] and 0.85–1.15 for PTEN (mean ± 2SD). Values below or above the respective cut-offs were considered as gene deletion or gene gain, respectively.

### Statistical analysis

Data on selected patient or tumor characteristics, prior treatments, response, skin toxicity, TTP following each treatment with cetuximab and survival were extracted from the records. TTP was estimated from initiation of each different line of treatment with cetuximab to the date progression of disease was documented. Survival was calculated from the date of diagnosis as well as from the first date of cetuximab treatment to the date of last contact or to the date of death from any cause. All survival probabilities were assessed according to the Kaplan-Meier method, whereas the log rank test was used to assess the effect of molecular markers on survival and TTP. Fisher's exact test was used to compare the results from IHC and FISH for the several molecular markers with respect to response and skin toxicity. All tests were two sided and a p-value of 0.05 or less was considered statistically significant. No multiple testing p-value adjustment was performed since this was only an exploratory analysis of retrospectively collected data in a very heterogeneously treated population.

### Ethics approval

The protocol was approved by the HeCOG Protocol Review Committee (HE R_6ER/05-22/10/2005) and by the Bioethics Committee of Aristotle University of Thessaloniki School of Medicine (A945-25/10/2005).

## Results

Seventy-two cetuximab treated patients (40 male and 32 female) were analyzed. Median age was 60 years. Most patients (52 of 72) had left sided (rectosigmoid) tumors and all except one had undergone surgery at the time of diagnosis. Duke's stage at diagnosis was: D for 39 (54%) of the patients; C for 19 (26%) and B for 12 (17%). Fifty-three patients (74%) had grade II disease. At the time of cetuximab therapy 54 patients (75%) had liver metastases and 31 (43%) had lung metastases. Other sites of metastases were lymph nodes, the abdomen and pelvis, bones, adrenals, brain, and spleen (Table 1). Ascites was recorded in 3 patients.

**Table 1:** Selected patient and tumor characteristics

**N**	**72**	
**Age**		
Median	60.1	
Range	29–76	
	
	**N**	**%**
	
**Sex**		
Male	40	56
Female	32	44
**Concurrent illness**		
No	44	61
Yes	28	39
**Initial Surgery**		
No	1	1
Yes	71	99
**Radical Operation**		
No	24	33
Yes	48	67
**Family history of neoplasia**		
No	47	65
Yes	22	31
Unknown	3	4
**Previous history of cancer**		
No	69	96
Yes	3	4
**Previous adjuvant treatment**		
No	46	64
Yes	24	33
Unknown	2	3
**Primary site**		
Cecum	6	8
Ascending	7	10
Transverse	5	7
Descending	2	3
Sigmoid	32	44
Rectum	20	28
**Stage**		
B1	1	1
B2	11	15
C1	3	4
C2	16	22
D	39	54
Unknown	2	3
**Histology grade**		
I	5	7
II	53	74
III	9	12
Unknown	5	7
**Metastatic sites of disease**		
Liver	54	75
Abdomen	8	11
Pelvis	3	4
Lung	31	43
Nodes	9	12
Bones	7	10
Adrenals	2	3
Serologic relapse	4	6
Other	4	6

Values were rounded up

Median follow-up was 53 months from diagnosis and 17 months from cetuximab initiation. At the time of the analysis 38 patients (53%) had died, most of them of disease. Two patients died of cardiovascular causes. Median overall survival from first colorectal cancer diagnosis was 46.8 months and from cetuximab initiation 14.9 months.

Cetuximab was given in combination with various regimens in all patients except one, who was treated with single agent cetuximab only. Two additional patients were treated with cetuximab alone after receiving it in combination with other regimens.

The most commonly used chemotherapeutic regimens were FOLFIRI in 27 patients, FOLFOX in 18 patients and irinotecan in 13 patients. A high percent of the patients (68%) were treated in second or third line, while 8.5% were treated in 1^st ^line, 15% in 4^th^, and 8.5% in 5^th ^line. Median time to progression (TTP) for patients treated in first to fourth line was between 6.2 and 7.6 months. Patients treated with cetuximab twice had a median TTP of 6.6 months the first time and 2.7 months the second time.

One patient achieved a complete response twice, both times in combination with irinotecan. Notably, this patient had lymph node only disease and very high EGFR amplification by FISH and normal PTEN. He progressed both times soon after stopping cetuximab maintenance and eventually died of disease.

62 patients were treated with cetuximab only in one line of therapy, while 10 were treated in two lines, for a total of 82 lines of treatment with cetuximab. Best response was CR in one patient, partial response (PR) in 23 and stable disease (SD) in 16 (two patients experienced CR and PR each, twice) (Table 2, Table 3). Skin toxicity (all grades) was encountered in 57 of 82 lines of treatment with cetuximab. Some degree of rash was seen in 21 of 24 lines of treatment leading to PR and in both lines of treatment in the patient who achieved CR, though grade 2 and 4 rash was only seen in 39 of 82 lines of treatment. Response to cetuximab was seen in 12.5% of patients with no rash versus 40% of patients who developed some degree of rash (p = 0.019).

**Table 2:** Response by line of treatment

	**Response**				
	
**Chemotherapy Line**	**CR**	**PR**	**SD**	**PD**	**NE**
**1^st^**	1	2	2	1	1
**2^nd^**	1	9	9	10	3
**3^rd^**	-	8	5	8	3
**4^th^**	-	4	1	5	2
**5^th^**	-	1	-	6	-

**Total**	2	24	17	30	9

**Table 3:** Response by treatment combination

	**Response**				
	
**Treatment combination**	**CR**	**PR**	**SD**	**PD**	**NE**
Cetuximab monotherapy	-	1	-	1	-
Cetuximab+FU+Leucovorin+CPT-11	-	11	7	7	2
Cetuximab+FU+Leucovorin+Eloxatin	-	7	2	8	1
Cetuximab+CPT-11+Eloxatin	-	-	1	1	2
Cetuximab+CPT-11+Capecitabine	-	-	1	2	1
Cetuximab+Eloxatin+Capecitabine	-	2	2	-	-
Cetuximab+CPT-11	2	2	2	7	-
Cetuximab+Eloxatin	-	-	-	-	1
	-	-	-	1	-
	-	-	1	-	-
Cetuximab+FU+CPT-11	-	1	-	-	-
Cetuximab+FU+Leucovorin	-	-	1	1	-
Cetuximab+CPT-11+Avastin	-	-	-	1	-
Cetuximab+Capecitabine	-	-	-	1	2

**Total**	2	24	17	30	9

Abbreviations: CR, complete response; PR, partial response; SD, stable disease; PD, progressive disease; NE, Non-evaluable.

### Biomarker expression and gene status analysis

**Figure 1:**
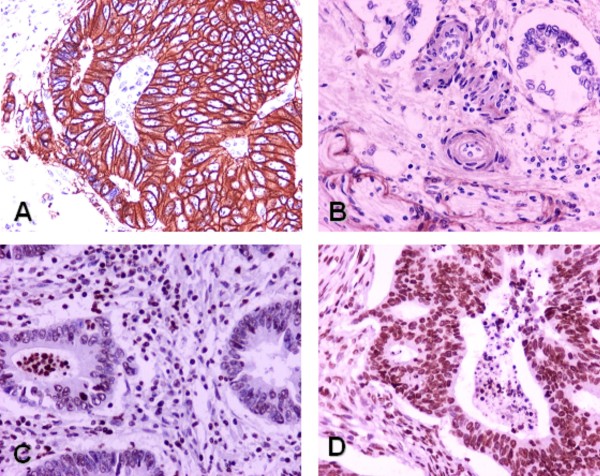
**Representative sections of immunohistochemical staining patterns (original magnification × 400)**. 1A. EGFR strong membrane staining (+3) in neoplastic cells; 1B. EGFR negativity in neoplastic cells, while in the perineurium the expression is not altered. 1C. PTEN protein loss in neoplastic cells, whereas the inflammatory cells showed strong nuclear staining; 1D. Intense PTEN protein expression in tumor cells.

A summary of the IHC and FISH findings are presented in Table 4. Membranous staining of EGFR protein expression was detected in 38/71 cases (54%), while over-expression (+2, +3) in 13 of the 38 EGFR-positive cases (34%). PTEN protein expression (no loss) was observed in 62/72 cases (86%) and the percentage of stained tumor cells in these cases ranged from 20% to over 90% (Figure 1). The majority of the patients showed PTEN nuclear staining, whereas nuclear and cytoplasmic staining was observed in 2 cases.

**Table 4:** Best response to treatment, and TTP on first line with cetuximab according to EGFR and PTEN status assessed by IHC and FISH

	**Number of patients**	**Response Rate**	**TTP on first line with cetuximab**
	**(N = 72)**	**N (%)**	**(months)**
**IHC**			
**EGFR**			
Negative	33	14 (42%)	6.85
Positive	38	10 (26%)	5.93
NE	1		
*P-value*			0.60
**PTEN**			
No loss	62	19 (31%)	6.39
Loss	10	5 (50%)	9.44
*P-value*			0.54
**FISH**			
**EGFR**			
Normal	56	20 (36%)	6.85
Gain	5	1 (20%)	8.72
Deletion	5	1 (20%)	6.39
NE	6		
*P-value*			0.18
**PTEN**			
Normal	43	18 (42%)	7.41
Gain	-	-	-
Deletion	23	3 (13%)	5.28
NE	6		
*P-value*			0.042

Abbreviations: NE, Non-evaluable, NRY, Not reached yet

**Figure 2:**
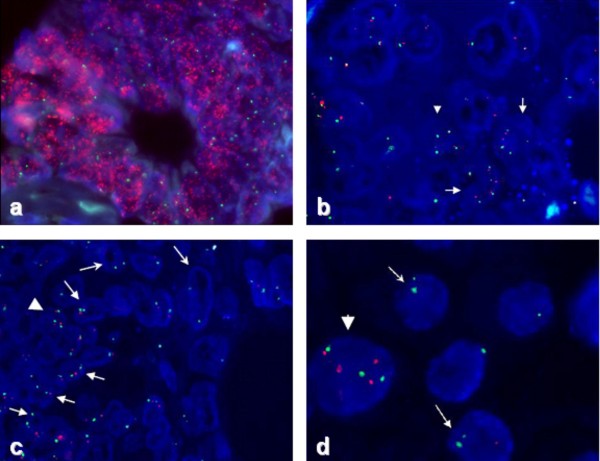
**Representative sections of gene expression by FISH**. a. High amplification of EGFR gene (red signals) in all tumor cells; b. Aneuploidy of chromosome 7 (green signals) accompanied by extra copies of EGFR gene (arrows). Five copies of chromosome 7 in one tumor cell are also visible (arrowhead); c. & d. Trisomy chromosome 10 (green signals) and extra copies of PTEN gene (red signals), (arrowhead), PTEN gene homozygous deletion (stealth arrow), heterozygous deletion (arrow).

EGFR gene status assessed by FISH, according to the criteria described above, was normal in 56/66 cases (85%). EGFR gene gain was observed in 5 cases, whereas gene deletion was seen in 5 cases, 4 of which were accompanied by lack of protein expression. PTEN gene deletion was observed in 23/66 cases (35%) (Figure 2).

No influence of EGFR expression as assessed by IHC and FISH or PTEN expression as assessed by IHC TTP and response to cetuximab was found.

**Figure 3:**
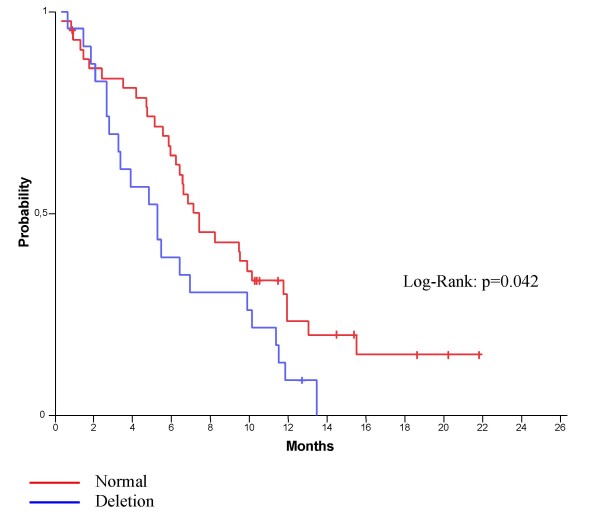
Kaplan-Meier curves for TTP according to PTEN expression (FISH).

More responses to cetuximab were seen in patients with wild type PTEN by FISH (see Tables 4 and 5). PTEN deletion was also associated with a significantly shorter TTP in the first line of treatment with cetuximab (deletion 5.28 months vs. normal 7.41 months, p = 0.042) (Figure 3).

**Table 5:** PTEN status (FISH) by chemotherapy line for responders (CR or PR)

	**PTEN**	
	
**Chemotherapy Line**	**Normal**	**Deletion**
1	1	1
2	7	1
3	7	1
4	4	0

**Total**	**19**	**3**

* In 3 patients one of which achieved PR twice PTEN status is unknown. One patient with normal PTEN achieved CR twice.

## Discussion

Although a number of EGFR targeted agents are approved for clinical use, in most cases the molecular determinants of response and resistance are not clear. EGFR is a transmebrane tyrosine receptor with kinase activity, which is over-expressed in many tumors. EGFR over-expression however, does not seem to be associated with response to and survival on cetuximab. In the present study we found that wild type PTEN was associated with response to and TTP on cetuximab. PTEN has been found to confer resistance to EGFR-inhibitors in glioblastoma, prostate, breast and non-small-cell lung cancer [15], and this resistance appears to be due to its function as a negative regulator of the phosphatidylinositol 3' kinase (PI3K) complex. PTEN inactivation leads to uncontrolled signaling trough the protein kinase B (PKB)/Akt pathway and to PIP3 accumulation, which in turn dissociates EGFR inhibition from the inhibition of the downstream signaling through this pathway. Whether inactivation of this pathway is sufficient for EGFR resistance is not clear. In a pre-clinical study it was shown that the induction of excess Akt in a PTEN normal, EGFR amplified cell line, alone, did not reverse sensitivity to an EGFR kinase inhibitor [21]. On the other hand, more recent in vitro studies support the significance of Akt accumulation in this setting [22].

In this study we investigated a segment of the colorectal cancer pathway, involved in the signaling of the EGFR pathway, in an effort to discover molecular determinants of cetuximab response. Due to the retrospective nature of the study the results are not definitive and should be approached with caution. In fact our group is analyzing tissue from additional patients for several molecular parameters and with more sophisticated techniques to confirm these results. Indeed many of the techniques used here are new and not standardized yet. Above all, these data need to be tested in a prospective study.

Clearly our patient population is heterogeneous in terms of combinations of previous chemotherapy received and line of chemotherapy associated with cetuximab. However this heterogeneity is consistent with current off-protocol practice in Greece. Furthermore, patients received cetuximab after failing the previous regimens and therefore could be considered resistant to them. This in turn indicates probable association of treatment effect with cetuximab use.

All these difficulties notwithstanding, it seems that we have identified a parameter that may be associated with cetuximab clinical benefit. The proposed mechanism, implicating PTEN mutations in EGFR resistance, is biologically plausible and has been described by other investigators in other malignancies [15]. More importantly, the recently published report by Frattini et al [10] also examined PTEN by IHC and discovered a correlation between loss of PTEN protein expression and lack of response to cetuximab. Even more recently another study examined the effect of cetuximab on several colon cancer cell lines and found that PTEN null, PIK3CA mutant and Ras/BRAF mutants cell lines are resistant to cetuximab. The authors even go as far as to recommend use of these parameters to stratify patients likely to benefit from cetuximab [23]. However, since wild type PTEN is quite common (60–100%) in colorectal adenocarcinoma, if it were the sole determinant of cetuximab response one would expect much higher response rates on cetuximab. Therefore it is more likely that PTEN is only one of the parameters that determine response to this therapeutic monoclonal antibody.

Whether PTEN is indeed a significant determinant of EGFR inhibitor efficacy must be investigated further and confirmed. Moreover, an optimal testing algorithm for the determination of PTEN status must be defined and standardized, so that it can be used in identifying patients who may be candidates for anti EGFR therapy. Finally, we will have to assess the significance of the co-overexpression of other molecules, such as MSI, VEGFR and K-RAS, as well as other downstream molecules of the EGFR pathway. Lastly, PTEN may also be inactivated via methylation and this should also be explored further [24].

## Conclusion

In conclusion, our clinical findings support previous data showing that the moAb cetuximab is an effective therapeutic agent against colorectal cancer. The molecular mechanisms of this response are complex, and probably dependent upon the expression levels of several signaling proteins of the EGFR and other pathways. PTEN, as suggested by our findings, seems to be particularly significant in this process.

## Competing interests

The authors declare that they have no competing interests.

## Authors' contributions

ER conceived of the study and participated in its design, in the recruitment of patients, in the collection and assembly of data and in the manuscript writing and final approval, EB conceived of the study and participated in its design, in the recruitment of patients and in the writing of the manuscript, EV provided paraffin blocks with tumor tissue and prepared and carried out the IHC/FISH assessments, DVS conceived of the study and participated in its design and in the recruitment of patients, DP conceived of the study and participated in its design and provided paraffin blocks with tumor tissue, IK provided paraffin blocks with tumor tissue, prepared and carried out the IHC/FISH assessments and participated in manuscript writing, ES conceived of the study and participated in its design and in the recruitment of patients, IX participated in the recruitment of patients, MBo provided paraffin blocks with tumor tissue, prepared and carried out the IHC/FISH assessments and participated in the manuscript writing, EG participated in the collection and assembly of data, MBa provided paraffin blocks with tumor tissue and prepared and carried out the IHC/FISH assessments, IG provided paraffin blocks with tumor tissue, AK provided paraffin blocks with tumor tissue, GK provided paraffin blocks with tumor tissue, PP conceived of the study and participated in its design and in the recruitment of patients, KTK conceived of the study and participated in its design, in the collection and assembly of data and in the manuscript writing and final approval, PK conceived of the study and participated in its design and in the recruitment of patients, GF conceived of the study and participated in its design, in the recruitment of patients, in the collection and assembly of data and in the manuscript writing and final approval.

All authors read and approved the final manuscript.

## Pre-publication history

The pre-publication history for this paper can be accessed here:


